# Prediction of amyloid PET positivity via machine learning algorithms trained with EDTA-based blood amyloid-β oligomerization data

**DOI:** 10.1186/s12911-022-02024-z

**Published:** 2022-11-07

**Authors:** Young Chul Youn, Hye Ryoun Kim, Hae-Won Shin, Hae-Bong Jeong, Sang-Won Han, Jung-Min Pyun, Nayoung Ryoo, Young Ho Park, SangYun Kim

**Affiliations:** 1 Department of Neurology, Chung-Ang University College of Medicine, Seoul, 06973 Republic of Korea; 2 Department of Neurology, Seoul National University College of Medicine and Clinical Neuroscience Center, Seoul National University Bundang Hospital, Seongnam-si, Gyeoggi-do 13629 Republic of Korea; 3 Department of Laboratory Medicine, Chung-Ang University College of Medicine, Seoul, 06973 Republic of Korea; 4 Department of Neurology, Soonchunhyang University Seoul Hospital, Soonchunhyang University College of Medicine, Seoul, 04401 Republic of Korea; 5 Department of Neurology, The Catholic University of Korea Eunpyeong St. Mary’s Hospital, Seoul, 03312 Republic of Korea; 6 Department of Medical Informatics, Chung-Ang University College of Medicine, Seoul, 06973 Republic of Korea

**Keywords:** Machine learning, Oligomer, Amyloid ß, Alzheimer’s disease, Biomarker, Multimer detection system, Amyloid positron emission tomography

## Abstract

**Background:**

The tendency of amyloid-β to form oligomers in the blood as measured with Multimer Detection System-Oligomeric Amyloid-β (MDS-OAβ) is a valuable biomarker for Alzheimer’s disease and has been verified with heparin-based plasma. The objective of this study was to evaluate the performance of ethylenediaminetetraacetic acid (EDTA)-based MDS-OAβ and to develop machine learning algorithms to predict amyloid positron emission tomography (PET) positivity.

**Methods:**

The performance of EDTA-based MDS-OAβ in predicting PET positivity was evaluated in 312 individuals with various machine learning models. The models with various combinations of features (i.e., MDS-OAβ level, age, apolipoprotein E4 alleles, and Mini-Mental Status Examination [MMSE] score) were tested 50 times on each dataset.

**Results:**

The random forest model best-predicted amyloid PET positivity based on MDS-OAβ combined with other features with an accuracy of 77.14 ± 4.21% and an F1 of 85.44 ± 3.10%. The order of significance of predictive features was MDS-OAβ, MMSE, Age, and APOE. The Support Vector Machine using the MDS-OAβ value only showed an accuracy of 71.09 ± 3.27% and F−1 value of 80.18 ± 2.70%.

**Conclusions:**

The Random Forest model using EDTA-based MDS-OAβ combined with the MMSE and apolipoprotein E status can be used to prescreen for amyloid PET positivity.

## Background

Alzheimer’s disease (AD) is a degenerative brain disease. It is associated with the loss of independent living due to the deterioration of cognitive function and is linked to the gradual loss of cortical neurons [1]. Amyloid beta (Aβ) plaques and neurofibrillary tangles are the pathological hallmarks of AD [2, 3]. The amyloid-β (Aβ) monomer is produced by β-secretase and γ-secretase from amyloid precursor protein, which is bound to the cell membrane. Aβ aggregates and forms multimers such as dimers, tetramers, hexamers, which are Aβ oligomers (AβOs). These multimers are the most toxic Aβ oligomers and have important roles in AD pathology [4]. They can further aggregate to form amyloid fibrils, which accumulate as amyloid plaques in the brain. Amyloid positron emission tomography (PET) imaging detects these fibrillary Aβ deposits [5].

Cerebral amyloidosis in AD is evaluated based on the CSF Aβ levels and amyloid PET imaging findings. However, these approaches are invasive and costly, which limits their clinical use [6].

Efforts have been made to develop blood-based Aβ-targeted biomarkers. The Multimer Detection System-Oligomeric Amyloid-β (MDS-OAβ) level is a valuable blood-based biomarker for AD. It is a modified sandwich immunoassay for measuring Aβ oligomerization in the plasma [7, 8]. This technique involves adding synthetic Aβ to the plasma to trigger oligomerization of Aβ to measure the oligomerization tendency of plasma Aβ in AD [9]. Since it is measured using plasma, the samples are treated with heparin or ethylenediaminetetraacetic acid (EDTA).

We previously evaluated the role of MDS-OAβ levels in heparin-treated plasma in differentiating between individuals with AD and community-based healthy participants [10]. This approach had high sensitivity and specificity. We also attempted to evaluate whether brain AD pathology could be predicted, based on blood MDS-OAβ levels in studies investigating the relationship between MDS-OAβ findings and magnetic resonance imaging or amyloid PET findings [11, 12].

Our previous studies have demonstrated MDS-OAβ cut-off levels and sensitivity and specificity values for clinical AD diagnosis. MDS-OAβ is a test that measures the dynamics of amyloid oligomerization in the blood, whereas amyloid PET detects static pathologies such as fibrillar Aβ plaques. Amyloid PET has been used as a standard biomarker for participant selection in many clinical trials. Mofrad et al. demonstrated a sensitivity of 76% and specificity of 67% for predicting amyloid PET positivity by using plasma MDS-OAβ and this technique reduced the costs and number of PET scans needed to screen for amyloidosis [13]. In this study, we tested various machine learning models to predict amyloid PET positivity using EDTA-based MDS-OAβ values combined with other variables (apolipoprotein E [APOE] genotype, age, and Mini-Mental Status Examination [MMSE] score).

In clinical AD prediction, MDS-OAβ has been validated and commercialized using heparinized plasma, and previous studies have used these samples [10]. However, most clinical centers store blood samples as EDTA-treated plasma. It is more accessible in clinical practice.

The objective of this study was to predict amyloid PET positivity based on EDTA-based blood Aβ oligomerization tendency to predict amyloid positivity by common machine learning algorithms in patients with memory complaints.

## Methods

### Study participants

This study was an observational cross-sectional study to evaluate machine learning models in predicting amyloid PET positivity of Aβ oligomerization tendency in which various features were used without previously provided cut-off values to predict the clinical decisions. This study was based on data obtained from the Alzheimer’s Disease All Markers Study (ADAM), which is a clinical study on protein biomarker development and early diagnosis of Alzheimer’s disease.

**Fig. 1:**
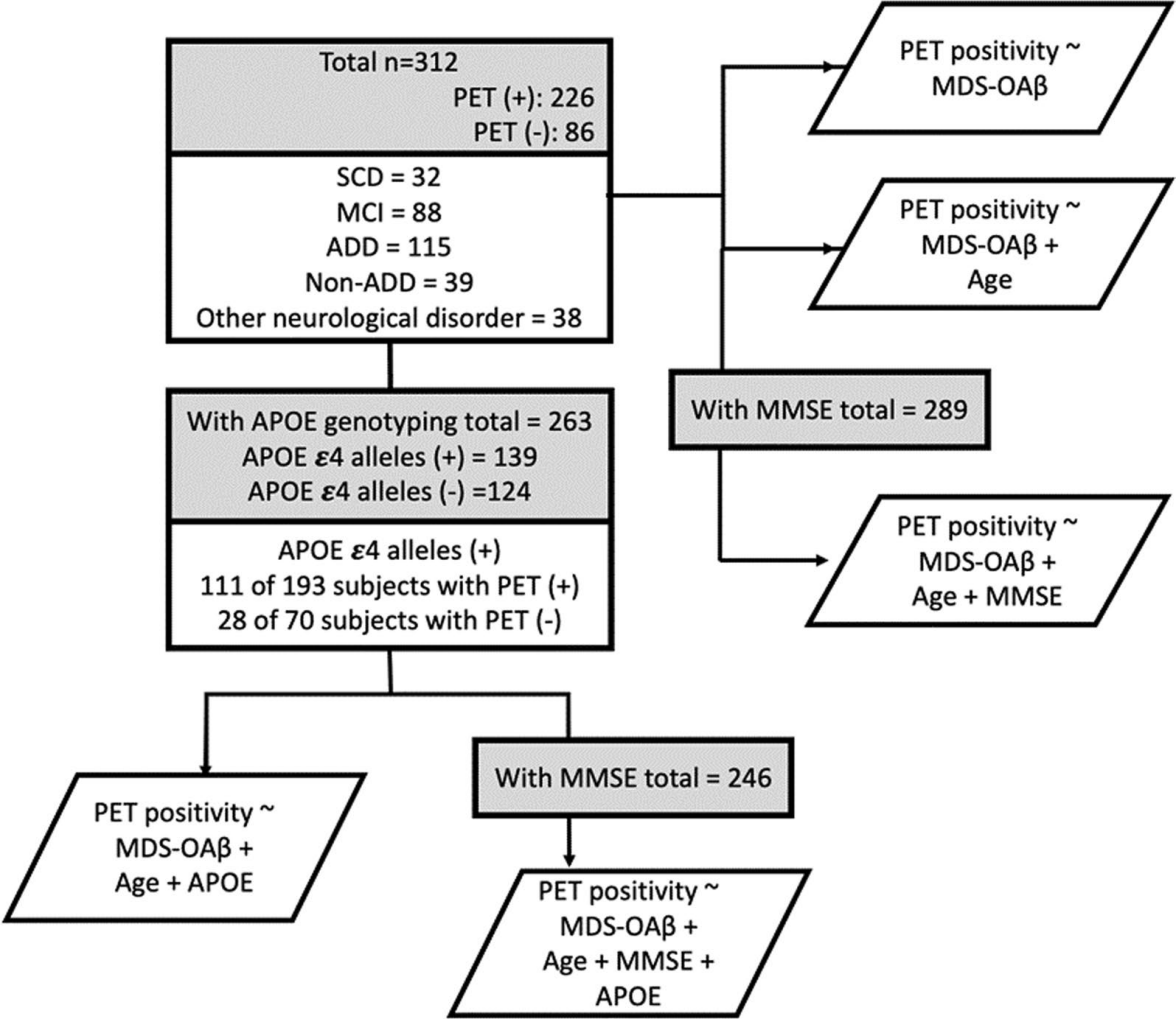
The enrollment of subjects for each machine learning model. SCD, Subjective Cognitive Decline; MCI, Mild Cognitive Impairment; ADD, Alzheimer’s Disease Dementia; Non-ADD, Non-Alzheimer type Dementia; MDS-OAβ, Multimer Detection System-Oligomeric Amyloid-β; MMSE, Mini-Mental Status Examination; APOE, apolipoprotein E

The participants were 312 patients who complained of memory abnormalities and had undergone EDTA-based MDS-OAβ and amyloid PET (Fig. 1). In addition to MDS-OAβ and amyloid PET, the MMSE was administered to 289 patients, and apolipoprotein E (APOE) genotypes were determined in 263 patients. The 312 participants included patients with subjective cognitive decline (n = 32), mild cognitive impairment (MCI; n = 88), AD dementia (n = 115), non-AD dementia (n = 39), and other neurological disorders (n = 38) such as alcohol-related cognitive impairment, parkinsonism, or individuals with postponed diagnosis.

The amyloid PET ligands used in this study were [^18^F]florbetaben (n = 286), [^18^F]flutemetamol (n = 24), and [^11^C]Pittsburgh compound B (n = 2). Amyloid PET status was defined as “positive” or “negative” which was assessed based on visual ratings from 3 different amyloid PET tracers by one nuclear medicine physician and one neurologist who were experienced and trained. They were blinded to the clinical information but knew the PET tracer utilized for each image. When there were discordances, the raters discussed and reached a consensus.Amyloid PET positive and negative findings were found in 226 patients and 86 patients, respectively (Table 1). The whole dataset used in this study is accessible via the following link: (https://drive.google.com/file/d/1XvMDK1OBsSiIxh4QlMQJbuLeqMmbMleA/view?usp=drivesdk; in the Gender column, “0” indicates female and “1” indicates male; in the PET column, “1” indicates positive and “0” indicates negative; “EDTA_MDS” represents the value of EDTA-based MDS-OAβ; in the APOE column, “0” indicates without the ε4 allele and “1” indicates with the ε4 allele; MMSE represents Mini-Mental Status Exam scores).

**Table 1:** Demographic characteristics of participants with amyloid PET positive and negative findings

Amyloid PET	Participants (n)		Men:Women (n:n)	Age (y) (mean ± SD)	EDTA-based MDS-OAβ value * (mean ± SD)	APOE 4 allele (no. of APOE 4 allele-positive patients/total no. of patients)	MMSE ** [mean score ± SD (no. of patients)]
Positive	226	87:139		68.4 ± 9.7	1.057 ± 0.242	111/193	17.9 ± 7.4 (213)
Negative	86	43:43		70.8 ± 8.8	0.972 ± 0.226	28/70	22.1 ± 5.6 (76)
Total	312	130:182		69.0 ± 9.5	1.034 ± 0.240	139/263	19.0 ± 7.3 (289)

Of the 312 participants, 263 participants were tested for apolipoprotein E (APOE) and 289 participants were administered the Mini-Mental Status Examination (MMSE). APOE was considered “positive” when at least one APOE epsilon 4 allele existed

PET, positron emission tomography; APOE, apolipoprotein E; SD, standard deviation; EDTA, ethylenediaminetetraacetic acid; MDS-OAβ, Multimer Detection System-Oligomeric Amyloid-β; MMSE, Mini-Mental State Examination

*The difference in MDS-OAβ values (*p*-value = 0.005)

**MMSE scores (*p*-value < 0.0001) between the amyloid PET positive and negative groups, based on the Student *t*-test

This study was approved by the institutional review board of the Seoul National University Bundang Hospital (Seongnam, Republic of Korea; approval number, B-2004-604-305). This study was conducted in accordance with the Declaration of Helsinki. The need for written informed consent was waived owing to the retrospective nature of the study.

### Data analysis and model training

We evaluated support vector machine (SVM), random forest (RF), logistic regression (LR), and deep neural network (DNN) machine learning models from scikit-learn (https://scikit-learn.org/stable/) [14] and TensorFlow (version 2.7.0; available at https://www.tensorflow.org) [15] to predict amyloid PET status using the EDTA-based MDS-OAβ levels combined with other variables. The scikit-learn is an open-source tool for predictive data analysis, and TensorFlow is also a commonly used open-source software library for machine learning developed by Google, Inc. (Mountain View, CA, USA), based on Python.

All the models were performed on the Colab (www.colab.research.google.com) cloud platform.

### Preprocessing

The total number of subjects for each model with the combination of variables were shown in Fig. 1. To model each algorithm, the dataset was subjected to the following preprocessing steps. Not all 312 subjects who underwent MDS-OAβ and amyloid PET did MMSE and APOE tests. Therefore, each model has a different number of subjects. We imported the data in the “.csv” format and dropped out cases having missing data.. Then we standardized features scaling to unit variance using ‘sklearn.preprocessing.StandardScaler’. The dataset was randomly split into the training dataset and test dataset. To split the dataset, we used “sklearn.model_selection.train_test_split”. The training data size was 70%, which indicated the percentage of the data to be withheld for training; the test dataset was thus composed of the remaining 30% of the data. In preprocessing, features and the outcome variable were created in each dataset.

### Model training

Each machine learning model based on various combinations of five variables: Age, APOE, EDTA-MDS, MMSE, and PET. APOE was “positive” if at least one APOE epsilon 4 allele existed and coded as “1”; otherwise, its value was set to 0. One variable, EDTA-MDS was the MDS-OAβ value, which was data obtained from EDTA anticoagulant plasma samples. The last variable, PET, which was included as the target outcome, was the “amyloid PET positivity.” Amyloid statuses were dichotomized as “positive” (coded as 1) or “negative” (coded as 0), based on visual assessment.

Because the dataset was obtained during clinical practice and was inevitably the data were disproportionate (participants had an amyloid PET positive to negative ratio of 226:86), this was mathematically compensated for using the ‘class_weight = 40:60’ parameter in all models.

The RF model used ‘ensemble.RandomForestClassifier’ with n_estimators = 1000 from sklearn tool. The following link will take users to the Python script for predicting amyloid positivity, given the values of EDTA-based MDS-OAβ levels and the other variables: https://colab.research.google.com/drive/1FzAgVcXJm9P2lssKPGqTD--QIfqKLU-6?usp=sharing. The SVM Classifier used ‘svm.SVM’ with decision boundary C = 3, the script is shown in following link: https://colab.research.google.com/drive/1hdzT7LIVIlX96FYwkA7kes186Ajk2NvD?usp=sharing. In the LR, linear_model.LogisticRegression was applied as following, https://colab.research.google.com/drive/1U_24Z15nPaMR7Q-YQlGvnw68JSfjVUhv?usp=sharing.

The model trained with a DNN consisted of one input, three hidden layers, and one output layer. The cost was calculated using “binary_crossentropy” and minimized using the “Adam” optimizer, of which script was following: https://colab.research.google.com/drive/1bILn1SvojLjViNaT9xIg69E-dB1aLa0b?usp=sharing.

To evaluate the performance, the accuracy, precision, recall and F1-value of amyloid PET prediction were calculated 50 times, using the randomly split test dataset with various combinations: “EDTA_MDS-OAβ”, “MMSE”, “EDTA_MDS-OAβ + MMSE”, “APOE”, “MDS-OAβ + MMSE + APOE”, and “MDS-OAβ + MMSE + Age + APOE.”.

In the dataset with multiple features, the features that contributed to the prediction accuracy of the machine learning algorithm showing the best performance were selected in order of contribution, and feature ranking with ‘recursive feature elimination’ was used (https://colab.research.google.com/drive/1qWuIf_Bql3gjNS-3gUnah2819ThlFWHv?usp=sharing).

The Student t-test was used to compare accuracy, precision, recall, and F1-value between groups. Differences were considered significant at *p* < 0.05.

## Results

Of the total 312 subjects with an MDS-OAβ, 289 had an MMSE score, 263 had APOE genotyping, and 246 patients had MDS-OAβ, MMSE, APOE, and age data (figure). Machine learning algorithms of SVM, LR, RF and DNN were performed on each data set. When comparing the mean value of the accuracies of the models with the statistical significance, the best performance was shown in the model using RF (Table 2). The RF model of amyloid PET prediction with EDTA-based MDS-OAβ was evaluated by training the “MDS-OAβ” feature alone or in combination with other features. As shown in Table 2, the accuracy, precision, recall and F1-value of ‘Age + MDS-OAβ + MMSE + APOE’ were 77.14 ± 4.21%, 80.75 ± 4.65%, 91.05 ± 4.78% and 85.44 ± 3.10%, which were better than those of MDS-OAβ value alone (*p* < 0.001). They were shown that the accuracy gradually increased as features were added. By using this dataset which had all MDS-OAβ, MMSE, APOE and Age feature data, features selection was conducted to determine the order of importance in predicting PET potential. The features that contribute to predicting amyloid PET positivity were in the order of MDS-OAβ, MMSE, Age and APOE.

**Table 2:** The mean performance of the MDS-OAβ predicting amyloid PET positivity, evaluated using various machine learning algorithms on 50 trials (mean ± standard deviation %)

Algorithms	Performance	MDS-OAβ	MDS-OAβ + Age	MDS-OAβ + Age + APOE	MDS-OAβ + Age + MMSE	MDS-OAβ + Age + MMSE + APOE
Subject number		N = 312	N = 312	N = 263	N = 289	N = 246
Support vector machine	Acc	71.09 ± 3.27**	69.21 ± 4.07	68.76 ± 3.99	68.69 ± 4.02	69.86 ± 4.82
	Prec	80.06 ± 4.46	76.70 ± 4.03	76.72 ± 4.50	78.25 ± 3.71	82.22 ± 5.25
	Rec	80.76 ± 5.38	83.13 ± 5.54	82.99 ± 4.80	78.93 ± 6.69	76.84 ± 5.68
	F1-value	80.18 ± 2.70	79.61 ± 3.05	79.59 ± 3.04	78.36 ± 3.45	79.24 ± 3.82
Random forest	Acc	66.08 ± 4.15	67.75 ± 3.61	69.49 ± 4.01	75.54 ± 3.98*	77.14 ± 4.21*†
	Prec	77.28 ± 4.61	75.68 ± 4.93	76.72 ± 5.54	79.84 ± 4.56	80.75 ± 4.65
	Rec	75.93 ± 5.57	82.17 ± 5.17	84.62 ± 4.56	89.81 ± 3.76	91.05 ± 4.78
	F1-value	76.40 ± 3.27	78.59 ± 3.08	80.26 ± 2.95	84.42 ± 2.92	85.44 ± 3.10
Logistic regression	Acc	69.13 ± 3.91**	69.00 ± 4.06	69.19 ± 4.98	69.38 ± 4.72	73.96 ± 5.30
	Prec	71.56 ± 3.78	73.33 ± 4.48	74.15 ± 5.27	75.59 ± 5.85	80.84 ± 5.21
	Rec	94.22 ± 3.76	90.30 ± 4.85	89.31 ± 7.41	86.56 ± 6.10	85.58 ± 5.99
	F1-value	81.25 ± 2.81	80.77 ± 2.93	80.72 ± 3.81	80.38 ± 3.35	82.94 ± 3.79
Deep neural network	Acc	64.00 ± 4.50	64.83 ± 4.45	64.50 ± 4.77	66.80 ± 5.16	69.24 ± 4.18†
	Prec	80.81 ± 4.90	77.19 ± 4.37	76.60 ± 4.82	78.50 ± 4.41	80.52 ± 4.12
	Rec	66.39 ± 8.25	74.20 ± 6.25	75.06 ± 6.31	75.65 ± 7.00	78.03 ± 6.18
	F1-value	72.46 ± 4.87	75.45 ± 3.64	75.61 ± 3.94	76.81 ± 4.04	79.03 ± 3.19

MDS-OAβ, Multimer Detection System-Oligomeric Amyloid-β; APOE, apolipoprotein E; Acc, accuracy; Prec, precision; Rec, recall

*p = 0.054, when compared ‘MDS-OAβ + Age + MMSE’ with ‘MDS-OAβ + Age + MMSE + APOE’

**p < 0.01, when compared ‘MDS-OAβ’ only of Support Vector Machine model with Logistic Regression

^†^*p* < 0.001, when compared ‘Random Forest’ with ‘Deep Neural Network’ algorithm based on the Student *t*-test

When only MDS-OAβ values were used, the SVM model showed the highest accuracy (71.09 ± 3.27) and was significantly better than LR (69.13 ± 3.91). For the SVM model, adding more features did not increase the accuracy. The accuracy of the DNN model was lower than other models across all datasets (64–69%).

## Discussion

The purpose of this study was to determine the accuracy of amyloid PET positive prediction regardless of the diagnosis using MDS-OAβ and to try algorithms with various feature combinations. Therefore, we used data from individuals with subjective cognitive decline, MCI, AD, and other neurodegenerative disorders.

The amyloid PET positive prediction accuracy of EDTA-based MDS-OAβ alone was 71.09 ± 3.27% using SVM model, and the accuracy with various feature combination using RF was 77.14 ± 4.21%, which was lower than our expectation at less than 80%.

However, when predicting positive amyloid PET by machine, the precision, which is the ratio of correctly predicted PET positive subjects to a total number of predicted positive PET, and the recall, which is the ratio between the numbers of PET positive subjects correctly predicted as positive to the total number of PET positives, were over about 80%. When it combined with Age, APOE, and MMSE features, the precision was 80.75% and the recall was 91.05%. In other words, as a screening tool, these machine learning algorithms using EDTA-based MDS-OAβ can be used to find brain amyloid pathologies.

There are several reasons that could explain why the performance did not meet expectations. First, the pathology or pathophysiology of Alzheimer’s disease reflected by MDS-OAβ and amyloid PET differs. The MDS-OAβ exhibits a tendency (i.e., dynamic change) of oligomerization of Aβ [9], whereas amyloid PET detects fibrillary Aβ plaques that have accumulated in the form of a sigmoid function graph since the onset of cerebral amyloidosis [16, 17]. Even in the early stages of AD dementia or an MCI state, amyloid plaques are already fully saturated in the brain [18]. However, the tendency of Aβ oligomerization is higher in the stage from MCI to early-stage AD dementia and decreases in a bell shape as it progresses to moderate to severe AD dementia [10]. A bell-shaped graph is obtained from the derivatives of the sigmoid function, which presents the MDS-OAβ value as a dynamic change in Aβ [19]. Therefore, the results of these two tests cannot be perfectly matched. EDTA-based MDS-OAβ reflects another aspect of the pathophysiology. However, it can be a tool that reflects brain amyloidosis in a different manner from amyloid PET in AD prediction.

Second, an expectation has been that cerebrospinal fluid (CSF) Aβ markers could be used to accurately predict brain amyloidosis because samples are obtained from closest to the brain in clinical practice and are regarded as reflecting brain pathology directly. However, CSF Aβ1-42 and amyloid PET findings were discordant in 21% of cognitively healthy people and 6% of dementia patients in one study [20]. Whether AD pathophysiology is caused by the centrifugal or centripetal spread of amyloidosis is not yet determined, but most researchers and clinicians believe that pathologic Aβ is produced in the brain and drains into CSF and bloodstream. More barriers, other than the CSF, exist in pathologic Aβ traveling from the brain to the blood, and the reflection of amyloid pathology is bound to be lower in the CSF.

Another consideration is diffuse Aβ plaques. Diffuse plaques are focal poorly marginated amyloid deposits that are not fibrillar (i.e., neuritic) and not associated with glial responses [21–23]. Some early indications were that Pittsburgh compound B (PIB), a PET tracer, binds to neuritic plaques but not to diffuse plaques [24]. The abundance of diffuse Aβ plaques can contribute to PET signals, although fibrillar Aβ is essential for higher PET tracer binding [25]. Plaques with more fibrillar amyloid have a greater affinity for Aβ ligands than do plaques with less fibrillar amyloid. A case of a mismatch between peripheral markers and amyloid PET has been reported, which demonstrated that amyloid PET findings may be negative but CSF Aβ may be decreased in AD with diffuse Aβ plaques [26].

Finally, a negative amyloid PET finding does not mean that the brain is free of Aβ plaques. Signals above the set threshold must be acquired to be interpreted as amyloid PET positive. Therefore, a negative finding should be regarded as meaning “not exceeding the set threshold” and that the presence of Aβ plaques in the brain cannot be completely ruled out.

Pyun et al.[11] statistically predicted amyloid PET positivity based on MDS-OAβ values using heparin plasma and showed higher accuracies. In the current study using EDTA plasma, the predictive accuracy of the machine learning algorithm using MDS-OAβ only was approximately 71.09%, and when the “MMSE” and “APOE” features were added, the accuracy was 77.14%. Heparin-based MDS-OAβ would be better in prescreening amyloid PET positive individuals. To predict clinical AD dementia, the validated decision cut-off value of MDS-OAβ is different depending on anticoagulant, EDTA-based MDS-OAβ values was approximately 1.0 ng/mL and the corresponding heparin-based level was 0.76 ng/mL. Plasma treated with different anticoagulants would have different mechanisms to obtain the values of MDS-OAβ. Heparin-based MDS-OAβ has been relatively well-validated than EDTA-based. Considering the accessibility of blood samples, however, EDTA-based MDS-OAβ may be an alternative for screening purposes. A comparative study of heparin-based MDS-OAβ versus EDTA-based MDS-OAβ is needed regarding their prescreening value for amyloid PET positivity.

When the “MMSE” and “APOE” features were concurrently included with the “MDS-OAβ” value, the performance of the algorithm significantly improved (*p* < 0.001). The ‘recursive feature elimination’ showed that the order of significance in contributing features was MDS-OAβ, MMSE, Age and APOE, and the MDS-OAβ is the most important feature in predicting amyloid PET positivity. The “Age” feature did not contribute to significant changes in predictive performance.

The APOE epsilon 4 allele is associated with amyloid PET [27, 28]. In this machine learning algorithm (data not shown), for “APOE” alone, the global predictive accuracy was less than that of “MDS-OAβ”; the variability of repeated measures was instead greater. APOE alone is insufficient. However, adding “MDS-OAβ” and “MMSE” to the “APOE” feature had an additive effect on the accuracy of predicting amyloid PET positivity in RF model.

One limitation of this study is the use of imbalanced data; in particular, 72.4% of the participants were amyloid PET-positive. Owing to the small number of participants, creating a random balance dataset to develop an algorithm that could be used to evaluate prediction accuracy was not possible. Datasets obtained in a clinical setting are unlikely to be balanced. The imbalance observed in this study was deemed acceptable; however, the presented findings must be interpreted with caution. Our previous study [29] did not show any differences in the accuracy of classification between imbalanced clinical datasets and randomly selected balanced datasets. Another limitation of this study is its retrospective design, whereby PET examinations were conducted at the discretion of the attending neurologist rather than using a standardized protocol.

## Conclusions

Machine learning algorithms to predict amyloid PET positivity performed satisfactorily when using the EDTA-based MDS-OAβ values as a predictive feature. The random forest model performed the best when using the MDS-OAβ combined with MMSE and APOE status, with the MDS-OAβ being the most predictive feature. The support vector machine model showed acceptable performance with MDS-OAβ as a single predictive feature. Machine learning models that use EDTA-based MDS-OAβ can be used to screen patients for amyloid PET positivity to predict those at risk of developing AD. The data were obtained retrospectively, and further well-designed prospective studies using balanced datasets are required to confirm the predictive value of EDTA-based MDS-OAβ for amyloid PET positivity..

## Data Availability

Some of the data were obtained from the Alzheimer’s Disease All Markers Study (ADAM). The whole dataset used in this study is accessible via the following link: https://drive.google.com/file/d/1XvMDK1OBsSiIxh4QlMQJbuLeqMmbMleA/view?usp=sharing.

## Abbreviations

AD: Alzheimer’s disease
ADAM: Alzheimer's disease all markers study
DNN: Deep neural network
EDTA: Ethylenediaminetetraacetic acid
LR: Logistic regression
MDS-OAβ: Multimer detection System-Oligomeric amyloid-β
PET: Positron emission tomography
RF: Random forest
SVM: Support vector machine
